# The Psychological Effect of COVID-19 on Home-Quarantined Nursing Students in China

**DOI:** 10.3389/fpsyt.2021.652296

**Published:** 2021-03-26

**Authors:** Dandan Li, Li Zou, Zeyu Zhang, Pu Zhang, Jun Zhang, Wenning Fu, Jing Mao, Shiyi Cao

**Affiliations:** ^1^School of Public Health, Tongji Medical College, Huazhong University of Science and Technology, Wuhan, China; ^2^Department of Neurology, Renmin Hospital of Wuhan University, Wuhan, China; ^3^Department of Neurology, Taihe Hospital, Hubei University of Medicine, Shiyan, China; ^4^Department of Cardiology, Taihe Hospital, Hubei University of Medicine, Shiyan, China; ^5^Department of Endocrinology, Taihe Hospital, Hubei University of Medicine, Shiyan, China; ^6^School of Nursing, Tongji Medical College, Huazhong University of Science and Technology, Wuhan, China; ^7^Key Laboratory of Emergency and Trauma, Ministry of Education, College of Emergency and Trauma, Hainan Medical University, Haikou, China

**Keywords:** anxiety, depression, post-traumatic stress symptoms, COVID-19, nursing students, China

## Abstract

Coronavirus disease 2019 (COVID-19) has significantly caused socioeconomic impacts. However, little is known about the psychological effect of COVID-19 on home-quarantined nursing students. The present study aimed to identify the prevalence and major determinants of anxiety, depression and post-traumatic stress symptoms (PTSS) in Chinese nursing students during the COVID-19 pandemic quarantine period. An online survey was conducted on a sample of 6,348 home-quarantined nursing students. Mental health status was assessed by the Generalized Anxiety Disorder 7-Item Scale (GAD-7), the Patient Health Questionnaire 9-Item Scale (PHQ-9) and the Post Traumatic Stress Disorder Check List-Civilian version (PCL-C), respectively. Logistic regression analyses were performed to identify risk factors of anxiety, depression and PTSS. The overall prevalence of anxiety was 34.97%, and the rates of “mild,” “moderate,” and “severe” anxiety were 26.24, 7.04, and 1.69%, respectively. Depression was detected in 40.22% of the nursing students, and the prevalence of “mild,” “moderate,” “moderately severe,” and “severe” depression was 27.87, 7.18, 4.08, and 1.09%, respectively. The overall prevalence of PTSS was 14.97%, with the prevalence of “mild” and “moderate-to-severe” PTSS reported at 7.04 and 7.93%, respectively. Male gender and insufficient social support were common risk factors for anxiety, depression and PTSS. In conclusion, about one-third, two-fifths, and one-seventh of Chinese nursing students had anxiety, depression and PTSS during the period of home quarantine, respectively. Timely and appropriate psychological interventions for nursing students should be implemented to reduce the psychological harm caused by COVID-19 pandemic.

## Introduction

Coronavirus disease 2019 (COVID-19) is a respiratory infectious disease caused by the severe acute respiratory syndrome coronavirus 2 (SARS-CoV-2), which was first detected in early December 2019 in Wuhan, China (1). As a major public health emergency, China defines COVID-19 as a category B infectious disease, and adopts the prevention and control measures of category A infectious disease. At present, China has achieved periodical results in the prevention and control of COVID-19, but the situation is still serious due to the increase of imported cases and asymptomatic cases.

As of April 18, 2020, novel coronavirus has affected more than 2 million individuals, and caused nearly 150,000 deaths worldwide. In addition to causing physical damage, COVID-19 also affects the mental health of the public. One study found that the rates of mental health symptoms among Chinese general population during the COVID-19 pandemic were 27.9% for depression and 31.6% for anxiety (2). A recent meta-analysis including 21 psychological studies showed that during this pandemic, mental health problems such as fear, anxiety and depression are common among the medical isolation population, patients with COVID-19 and front-line medical staff (3). However, researches on the psychological status of nursing students undergoing long-term home quarantine were limited. Nursing students are an important part to promote the sustainable development of the medical industry. Healthy psychology is crucial for them to complete their studies and be competent for clinical work. Individuals being in quarantine may experience psychological distress in the form of anxiety, confusion and stress symptoms (4). In addition, many previous studies showed that psychological problems of medical students may affect the choice of medical career and even lead to students' suicide (5–7). Currently, only several studies have reported nursing students' sleep quality and their stress levels before and during lockdown due to the COVID-19 pandemic (8–10).

In China, university students have left school since mid-January 2019 and been quarantined at home because of the COVID-19 pandemic. Until the end of this investigation, no students were allowed to return to school, and the government or colleges did not tell them when the new term began. To our knowledge, studies regarding psychological status and related risk factors among home-quarantined nursing students in China are still lacking. Therefore, the aim of the present study was to estimate the prevalence of anxiety, depression and post-traumatic stress symptoms (PTSS) and identify the associated factors in Chinese nursing students during the COVID-19 pandemic quarantine period. The findings would contribute to formulate effective interventions on psychological health, so as to improve the mental health level of nursing students.

## Methods

### Ethics Statement

The study was approved by the Research Ethics Committee in Tongji Medical College, Huazhong University of Science and Technology, Wuhan, China (IORG0003571).

### Participants and Sampling

This cross-sectional survey was conducted from March 8, 2020, to March 24, 2020. We selected 18 colleges using a convenient sampling method, and recruited nursing students in each college to participate in this survey. The inclusion criteria for the participants were: (1) full-time nursing students, and (2) willingness to participate in this study. The exclusion criterion was: (1) those with a history of past mental illness diagnoses. Data were collected through Questionnaire Star (https://www.wjx.cn) with an anonymous, self-rated questionnaire that was distributed to all selected colleges over the internet. All participants provided informed consent electronically prior to registration. The informed consent page presented two options (yes/no). Only participants who chose “yes” were taken to the questionnaire page. The online questionnaire was distributed to 6,500 nursing students. Finally, 6,348 students responded, with a response rate of 97.66%.

### Measurement

Anxiety was measured using the Generalized Anxiety Disorder 7-Item Scale (GAD-7) (11). The scale consists of seven items asking the respondents how often, during the period of home isolation, they were bothered by each symptom. For example, “Feeling nervous, anxious, or on edge.” The answer options were “not at all,” “several days,” “more than half the days,” and “nearly every day” scored from 0 to 3 points. Possible range of scores is from 0 to 21, with the higher scores indicating the presence of more symptoms. The GAD score, based on the severity of anxiety symptoms, is categorized as “no anxiety” = 0–4, “mild anxiety” = 5–9, “moderate anxiety” = 10–14, and “severe anxiety” = 15–21 (11, 12). In this study, Cronbach's alpha for the scale was 0.93, indicating good internal consistency.

Depression was assessed using the Patient Health Questionnaire 9-Item Scale (PHQ-9) (13). The PHQ-9 contains nine items asking the respondents how often they were bothered by each symptom during the period of home isolation. For instance, “Little interest or pleasure in doing things.” Response options included “not at all,” “several days,” “more than half the days,” and “nearly every day” scored from 0 to 3 points. The maximum score of PHQ-9 is 27 points, and a minimum score is 0 points. The scores are classified as: 0–4 (no depression), 5–9 (mild depression), 10–14 (moderate depression), 15–19 (moderately severe depression), and 20–27 (severe depression) (13). In the present study, the PHQ-9 demonstrated high internal consistency (Cronbach's α = 0.93).

PTSS was measured using the Post Traumatic Stress Disorder Check List-Civilian Version (PCL-C) (14). The scale consists of 17 items asking the respondents how much they had been bothered by a symptom during the period of home isolation. For example, “Feeling jumpy or easily startled?” Each item is scored on a five-point Likert scale, ranging from “not at all” to “extremely” coded with values from 1 to 5. Total scores range from 17 to 85, with the higher scores indicating the presence of more symptoms. The score of PCL-C is categorized as “no PTSS” = 17–37, “mild PTSS” = 38–49 and “moderate to severe PTSS” = 50–85 (15). Cronbach's alpha for the PCL-C was 0.95 in this study.

To identify the factors which may be associated with nursing students' mental health, information on demographic characteristics (gender, grade, residence, self-perceived family economic status, exercise status during the COVID-19 pandemic, whether you are the only-child or not, whether participate in clinical practice in the past, whether your parents are medical personnel or not) and social support was collected.

Social support was measured using the Multidimensional Scale of Perceived Social Support (MSPSS) (16). The scale contains 12 items scored on a seven-point Likert scale ranging from 1 (strongly disagree) to 7 (strongly agree), measuring the extent to which each item was experienced. MSPSS has three dimensions as family, friend, and special person support which represent the support sources. Each dimension involves 4 items. The 3, 4, 8, and 11 items measure the family support, 6, 7, 9, and 12 items measure friend support, and 1, 2, 5, and 10 items measure a special person's support (17). The MSPSS score of 12–36 suggests “low-level social support,” 37–60 suggests “medium-level social support,” whereas 61–84 suggests “high-level social support” (18). Cronbach's alpha for the scale was 0.96 in this study.

### Statistical Analysis

All analyses were performed using the Statistical Analysis System (SAS) 9.4 for Windows (SAS Institute Inc., Cary, NC, USA). Participants' sociodemographic characteristics and the levels of anxiety, depression and PTSS were described using frequency and percentage. Cutoff scores of 5 for the GAD-7 (2), 5 for the PHQ-9 (2), and 38 for the PCL-C were adopted to detect probable symptoms of anxiety, depression, and PTSS for all remaining analyses (19). The Chi-square test was conducted to compare the prevalence of anxiety, depression and PTSS across groups defined by demographic data and social support levels. Where significant differences were noted, Phi/Cramer's V was used to measure the magnitude of the differences. Three separate logistic regression models, where the dependent variables were anxiety, depression, and PTSS, were performed to identify the associated factors. All comparisons were two-tailed, and *p*-values < 0.05 were considered statistically significant.

## Results

### Sociodemographic Characteristics of Respondents

Participant characteristics are presented in Table 1. The majority of the respondents (90.37%) were females, and 35.66% resided in urban areas. Most respondents were juniors (30.01%), followed by freshmen (27.80%), and sophomores (25.41%). A good self-perceived family economic status was reported by 6.68% of the participants, while 22.89% reported poor economic status. Approximately 40% of the nursing students exercised regularly during the COVID-19 pandemic. Less than half of the students (48.90%) had participate in clinical practice in the past.

**Table 1:** Sociodemographic characteristics of respondents.

**Characteristic**	***N***	**%**
**Gender**		
Male	611	9.63
Female	5,737	90.37
**Grade**		
Freshman	1,765	27.80
Sophomore	1,613	25.41
Junior	1,905	30.01
Senior	920	14.49
Intern	56	0.88
Postgraduate	89	1.40
**Residence**		
Urban	2,264	35.66
Rural	4,084	64.34
**Self-perceived family economic status**		
Good	424	6.68
Fair	4,471	70.43
Bad	1,453	22.89
**Exercise status during the COVID-19 pandemic**		
Exercise regularly	2,376	37.43
Lack of exercise	3,972	62.57
**Whether you are the only-child or not**		
Yes	1,579	24.87
No	4,769	75.13
**Whether participate in clinical practice in the past**		
Yes	3,104	48.90
No	3,244	51.10
**Whether your parents are medical personnel or not**		
Yes	193	3.04
No	6,155	96.96

### Prevalence of Anxiety, Depression, and PTSS

The overall prevalence of anxiety was 34.97% (2,220/6,348), among which the prevalence of “mild,” “moderate,” and “severe” anxiety was 26.24, 7.04, and 1.69%, respectively. The overall prevalence of depression was 40.22% (2,553/6,348), and the prevalence of “mild,” “moderate,” “moderately severe,” and “severe” depression was 27.87, 7.18, 4.08, and 1.09%, respectively. The overall prevalence of probable PTSS was 14.97% (950/6,348), with the prevalence of “mild” and “moderate-to-severe” PTSS reported at 7.04 and 7.93%, respectively (Table 2).

**Table 2:** Prevalence of anxiety, depression and PTSS at different levels among nursing students.

**Scale**	**Categories**	***N***	**%**
GAD-7^a^			
	No anxiety	4,128	65.03
	Mild anxiety	1,666	26.24
	Moderate anxiety	447	7.04
	Severe anxiety	107	1.69
PHQ-9^b^			
	No depression	3,795	59.78
	Mild depression	1,769	27.87
	Moderate depression	456	7.18
	Moderately severe depression	259	4.08
	Severe depression	69	1.09
PCL-C^c^			
	No PTSS	5,398	85.03
	Mild PTSS	447	7.04
	Moderate to severe PTSS	503	7.93

a *GAD-7, Generalized Anxiety Disorder 7-Item Scale*.

b *PHQ-9, Patient Health Questionnaire 9-Item Scale*.

c *PCL-C, Post Traumatic Stress Disorder Check List – Civilian version*.

The prevalence of anxiety, depression and PTSS was significantly higher in males than in females. The rates of anxiety and depression in nursing students lacking of physical exercise were significantly higher than those in students who exercised regularly during the COVID-19 pandemic. Compared with nursing students without undergoing clinical practicum, those who had participated in clinical practice in the past had a higher rate of anxiety. Nursing students who reported high-level social support had lower prevalence of anxiety, depression and PTSS compared to those with middle-level and low-level social support. More information is showed in Table 3.

**Table 3:** Anxiety, depression, and PTSS among nursing students with different sociodemographic characteristics and social support levels.

**Characteristic**	**Total *N***	**Anxiety*n* (%)**	***P*-value**	**Phi/Cramer's V**	**Depression *n* (%)**	***P*-value**	**Phi/Cramer's V**	**PTSS^†^*n* (%)**	***P*-value**	**Phi/Cramer's V**
**Gender**										
Male	611	241 (39.44)	0.0148	0.03	280 (45.83)	0.0029	0.04	160 (26.19)	<0.0001	0.10
Female	5,737	1,979 (34.50)			2,273 (39.62)			790 (13.77)		
**Grade**										
Freshman	1,765	552 (31.27)^a^	0.0021	0.05	697 (39.49)	0.8173	–	252 (14.28)	0.7317	–
Sophomore	1,613	574 (35.59)^*^			635 (39.37)			234 (14.51)		
Junior	1,905	688 (36.12)^b^			780 (40.94)			302 (15.85)		
Senior	920	343 (37.28)^b^			378 (41.09)			137 (14.89)		
Intern	56	25 (44.64)^*^			24 (42.86)			9 (16.07)		
Postgraduate	89	38 (42.70)^*^			39 (43.82)			16 (17.98)		
**Residence**										
Urban	2,264	757 (33.44)	0.0562	–	901 (39.80)	0.6109	–	316 (13.96)	0.0924	–
Rural	4,084	1,463 (35.82)			1,652 (40.45)			634 (15.52)		
**Self-perceived family economic status**										
Good	424	128 (30.19)^a^	<0.0001	0.07	136 (32.08)^a^	<0.0001	0.09	60 (14.15)^*^	<0.0001	0.06
Fair	4,471	1,503 (33.62)^a^			1,720 (38.47)^b^			611 (13.67)^a^		
Bad	1,453	589 (40.54)^b^			697 (47.97)^c^			279 (19.20)^b^		
**Exercise status during the COVID-19 pandemic**										
Exercise regularly	2,376	760 (31.99)	0.0001	0.05	807 (33.96)	<0.0001	0.10	345 (14.52)	0.4419	–
Lack of exercise	3,972	1,460 (36.76)			1,746 (43.96)			605 (15.23)		
**Whether you are the only-child or not**										
Yes	1,579	526 (33.31)	0.1106	–	606 (38.38)	0.0856	–	219 (13.87)	0.1590	–
No	4,769	1,694 (35.52)			1,947 (40.83)			731 (15.33)		
**Whether participate in clinical practice in the past**										
Yes	3,104	1,146 (36.92)	0.0015	0.04	1,260 (40.59)	0.5507	–	475 (15.30)	0.4609	–
No	3,244	1,074 (33.11)			1,293 (39.86)			475 (14.64)		
**Whether your parents are medical personnel or not**										
Yes	193	58 (30.05)	0.1455	–	79 (40.93)	0.8369	–	26 (13.47)	0.5546	–
No	6,155	2,162 (35.13)			2,474 (40.19)			924 (15.01)		
**Social support level**										
Low	118	55 (46.61)^a^	<0.0001	0.15	67 (56.78)^a^	<0.0001	0.19	32 (27.12)^a^	<0.0001	0.17
Medium	2,608	1,125 (43.14)^a^			1,316 (50.46)^a^			561 (21.51)^a^		
High	3,622	1,040 (28.71)^b^			1,170 (32.30)^b^			357 (9.86)^b^		

† *PTSS, post-traumatic stress symptoms*.

* *No significant differences in frequency/% compared to the rest categories after bonferonni correction*.

a, b, c *Different letters indicate significant differences in frequency/% after bonferonni correction; the same letter indicates no significant difference after bonferonni correction*.

### Influencing Factors of Anxiety, Depression, and PTSS

Table 4 presents the results of the multivariate logistic regression analysis, where the dependent variables were anxiety, depression and PTSS. Factors significantly associated with anxiety among nursing students included male gender (OR = 1.28, 95% CI: 1.08–1.53), bad family economic status (OR = 1.31, 95% CI: 1.03–1.67) and insufficient social support (OR = 2.06, 95% CI: 1.42–3.00 for low-level and OR = 1.83, 95% CI: 1.64–2.03 for medium-level). Compared to freshman, sophomore (OR = 1.23, 95% CI: 1.06–1.42), junior (OR = 1.20, 95% CI: 1.02–1.41) and senior (OR = 1.28, 95% CI: 1.04–1.57) had higher odds for anxiety. Respondents lacking of physical exercise were more likely to show anxiety compared to those who exercised regularly during the COVID-19 pandemic, and the OR was 1.14 (95% CI: 1.02–1.27).

**Table 4:** Multivariate logistic regression analysis of factors associated with anxiety, depression and PTSS among nursing students.

**Characteristic**	**Anxiety**			**Depression**			**PTSS^d^**		
	**OR^b^**	**95% CI^c^**	***P***	**OR**	**95% CI**	***P***	**OR**	**95% CI**	***P***
**Gender (Ref.^a^**=** Female)**									
Male	1.28	1.08–1.53	0.0057	1.32	1.11–1.58	0.0015	2.26	1.84–2.77	<0.0001
**Grade (Ref**. **=** **Freshman)**									
Sophomore	1.23	1.06–1.42	0.0060	1.00	0.87–1.16	0.9793	1.12	0.92–1.36	0.2780
Junior	1.20	1.02–1.41	0.0291	1.07	0.92–1.25	0.3929	1.24	1.00–1.54	0.0539
Senior	1.28	1.04–1.57	0.0197	1.12	0.92–1.37	0.2697	1.21	0.91–1.60	0.1874
Intern	1.57	0.90–2.74	0.1099	1.04	0.59–1.82	0.8928	1.13	0.53–2.40	0.7577
Postgraduate	1.57	0.99–2.47	0.0533	1.21	0.77–1.91	0.4152	1.42	0.78–2.56	0.2510
**Residence (Ref**. **=** **Rural)**									
Urban	0.97	0.86–1.09	0.6149	1.08	0.96–1.21	0.1804	0.97	0.82–1.13	0.6694
**Self-perceived family economic status (Ref**. **=** **Good)**									
Fair	1.06	0.85–1.32	0.6059	1.20	0.96–1.50	0.1042	0.90	0.82–1.13	0.4639
Bad	1.31	1.03–1.67	0.0298	1.66	1.30–2.11	<0.0001	1.18	0.86–1.63	0.3032
**Exercise status during the COVID-19 pandemic (Ref**. **=** **Exercise regularly)**									
Lack of exercise	1.14	1.02–1.27	0.0249	1.42	1.27–1.58	<0.0001	0.97	0.84–1.13	0.6928
**Whether you are the only-child or not (Ref**. **=** **No)**									
Yes	0.95	0.84–1.09	0.4768	0.92	0.82–1.05	0.2212	0.89	0.75–1.06	0.1822
**Whether participate in clinical practice in the past (Ref**. **=** **No)**									
Yes	1.07	0.93–1.23	0.3491	0.94	0.82–1.08	0.4031	0.97	0.80–1.17	0.7239
**Whether your parents are medical personnel or not (Ref**. **=** **No)**									
Yes	0.83	0.60–1.14	0.2573	1.09	0.80–1.47	0.5773	0.91	0.59–1.41	0.6761
**Social support level (Ref**. **=High)**									
low	2.06	1.42–3.00	0.0001	2.46	1.69–3.58	<0.0001	3.19	2.08–4.90	<0.0001
Medium	1.83	1.64–2.03	<0.0001	2.02	1.81–2.24	<0.0001	2.47	2.13–2.86	<0.0001

a *Ref, reference*;

b *OR, odds ratio*;

c *CI, confidence interval*;

d *PTSS, post-traumatic stress symptoms*.

Factors significantly associated with depression among nursing students included male gender (OR = 1.32, 95% CI: 1.11–1.58), bad family economic status (OR = 1.66, 95% CI: 1.30–2.11), and insufficient social support (OR = 2.46, 95% CI: 1.69–3.58 for low-level and OR = 2.02, 95% CI: 1.81–2.24 for medium-level). Compared with students who exercised regularly during the COVID-19 pandemic, those lacking of physical exercise had higher odds for depression (OR = 1.42, 95% CI: 1.27–1.58).

Respondents who were male (OR = 2.26, 95% CI: 1.84–2.77), and those who reported insufficient social support (OR = 3.19, 95% CI: 2.08–4.90 for low-level and OR = 2.47, 95% CI: 2.13–2.86 for medium-level) showed a higher likelihood of having PTSS.

## Discussion

The outbreak of COVID-19 in China has a direct or indirect impact on all areas of society. In order to curb the outbreak and protect students from COVID-19, all schools have been closed till the epidemic is under control. Students facing long-term home quarantine and online learning are prone to a series of stress emotional response such as a higher level of anxiety and other negative emotions (20). Our study assessed the prevalence of anxiety, depression and PTSS among home-quarantined Chinese nursing students and explored the related risk factors. The results suggested that the pandemic of COVID-19 had a certain impact on the psychology of Chinese nursing students.

In the present study, the prevalence of anxiety and depression was about 35 and 40%, respectively. Rates of mental health problems among nursing students were reported ranging from 13.8 to 26% for anxiety (21–24) and 21.2 to 56.4% for depression (21–25). Compared with studies conducted in a normal period (21, 22, 24, 25), a much higher rate of anxiety was observed in our study. Chang et al. (20) found that during the COVID-19 pandemic, the prevalence of anxiety was 26.6%, 21.2% for depression among college students, and pointed that the rate of mental health problems was related to students' professional background. Usually, medical students are more concerned about the COVID-19 and its further consequences. At early stages of this pandemic, people have little information about nature, treatment, fatality rate, etc., which could aggravate their fear about the infectious disease (26). With the rapid spread of COVID-19, students receiving a large amount of negative information is in more risk of psychological maladjustment (20).

Nursing is historically a female-dominated profession. However, increasing numbers of male students have chosen nursing major in recent decades, narrowing the gender gap. Our study found that the prevalence of anxiety, depression and PTSS in male nursing students was significantly higher than that in female nursing students. A study conducted by Ji et al. (23) showed that there was no significant gender difference in anxiety and depression rates among college students during the COVID-19 pandemic. In the present study, male nursing students had higher odds for anxiety, depression and PTSS, while a study conducted in Italian general population found that female gender was associated with higher levels of depression, anxiety, and stress (27). The reason may be attributable to biopsychosocial factors such as traditional beliefs, social prejudice, and professional characteristics, which may cause male nursing students to face great social pressure and psychological pressure. After the COVID-19 pandemic, further studies with larger samples are needed to verify whether male nursing students are at increased risk for mental health problems.

Social support is an important environmental resource for individuals in social life, and is closely related with the individual's mental health (28). An earlier study indicated that social support was an important variable that have been shown to be negatively associated with anxiety and depression among nursing students (24). In this study, nursing students with low-level and medium-level social support accounted for ~40%, and these students had higher risk for anxiety, depression and PTSS compared with students with high-level social support. Therefore, we should attach importance to the role of social support for maintaining students' mental health. On the one hand, parents should enhance communication with their children to give full play to the role of family psychological support. On the other hand, colleges should set up online mental health courses about the COVID-19 pandemic to improve the students' psychological adaptability.

Family economic status was an important influencing factor of anxiety and depression in our study. Nursing students who reported poor financial status were more likely to experience anxiety and depression than those who reported good family economic status. The finding is in line with previous studies. Teris et al. (29) found that nursing students in financial difficulties were 2.3 times and 2.6 times more likely to experience anxiety and depression than those without. Andrews and Wilding (30) showed that financial vulnerability may exacerbate anxiety and depression among university students. Other researchers found that higher family income was inversely associated with a lower prevalence of depression (31–35). In order to control the spread of COVID-19 pandemic, many companies and factories have postponed their operation, which inevitably affected the economic income of some families. Under such circumstances, it may be hard for students to maintain a healthy mentality.

In this study, sophomore, junior and senior were more likely to develop anxiety and depression than freshman. This may be related to the School of Nursing curriculum design. Freshmen are not required to undertake any clinical practicum. Exemption from the clinical practicum may relieve some anxiety, depression, and stress (29). Moreover, the academic pressure of high-grade students is greater, and some of them were facing graduation, employment, and clinical practice, etc., but the progress of various things is inevitably affected by the outbreak of COVID-19.

Compared with nursing students who exercised regularly during the pandemic of COVID-19, those lacking of physical exercise had higher odds for anxiety and depression. Similarly, a study conducted by Feng et al. (36) showed that physical inactivity was independently associated with a higher risk of depression and poor sleep. This may suggest that regularly physical exercise is a protective factor for students' mental health. However, university students usually spend long hours studying online during the period of home quarantine, which means they exercise less.

Several limitations of our study should be mentioned. First, since this was a cross-sectional study, causal relations between the presence of anxiety, depression, and PTSS and variables cannot be determined. Second, self-rating scales were used to assess anxiety, depression, and PTSS, thus response bias may exist. However, a face-to-face in-depth interview was impossible to conduct due to the whole country under lockdown. Third, most of the nursing students are female (23), therefore, the research results may not be extended to students in other majors. Fourth, knowledge and behaviors regarding the COVID-19 pandemic are important factors that may affect individual mental health. A study involving 2,125 Italian undergraduate students showed an acceptable level of knowledge regarding this pandemic and the control measures adopted (37). Our study did not investigate the knowledge and behaviors about the COVID-19 pandemic. Further research was needed to explore the impact of these factors on psychology. Fifth, we consulted experts on the used scales, but a pilot-test among nursing students was not conducted to evaluate the face validity of these scales.

In conclusion, about one-third, two-fifths, and one-seventh of Chinese nursing students had anxiety, depression, and PTSS during the COVID-19 pandemic quarantine period, respectively. Nursing students who were male, who reported bad family economic status, who obtained insufficient social support, and those lacking of physical exercise were more prone to psychological problems. The COVID-19 pandemic is still ongoing, and many students remain isolated at home. Timely and appropriate psychological interventions for nursing students should be implemented to reduce the psychological harm caused by the pandemic.

## Data Availability Statement

The raw data supporting the conclusions of this article will be made available by the authors, without undue reservation.

## Ethics Statement

The studies involving human participants were reviewed and approved by the Research Ethics Committee in Tongji Medical College, Huazhong University of Science and Technology, Wuhan, China.

## Author Contributions

SC and JM conceived and designed the study. WF participated in the acquisition of data. DL and LZ analyzed data and drafted the manuscript. ZZ, PZ, and JZ revised the manuscript. SC, JM, and WF are the guarantors of this work and have full access to all the data in the study and take responsibility for its integrity and the accuracy of the data analysis. All authors read and approved the final manuscript.

## Conflict of Interest

The authors declare that the research was conducted in the absence of any commercial or financial relationships that could be construed as a potential conflict of interest.
